# Case Report: Solitary Extramedullary Plasmacytoma in the Cervix Misdiagnosed as Cervical Cancer

**DOI:** 10.3389/fonc.2021.685070

**Published:** 2021-06-04

**Authors:** Ji Wang, Lin Jiang, Xuejin Ma, Tingchao Li, Heng Liu, Xiaoxi Chen, Shiguang Li

**Affiliations:** ^1^Department of Radiology, The First People’s Hospital of Zunyi, The Third Affiliated Hospital of Zunyi Medical University, Zunyi, China; ^2^Department of Pathology, The First People’s Hospital of Zunyi, The Third Affiliated Hospital of Zunyi Medical University, Zunyi, China; ^3^Department of Radiology, Medical Imaging Center of Guizhou Province, Affiliated Hospital of Zunyi Medical University, Zunyi, China

**Keywords:** solitary extramedullary plasmacytoma, solitary plasmacytoma, cervical cancer, MRI, case report

## Abstract

Solitary plasmacytoma (SP) is a malignant tumor caused by the monoclonal proliferation of plasma cells, representing less than 5% of plasma cell tumors. SP can be categorized into two groups: solitary bone plasmacytoma (SBP) and solitary extramedullary plasmacytoma (SEP). SEP most commonly occurs in the head and neck and is rarely located in the reproductive system. Here, we report a case of a 77-year-old woman with SEP in the cervix who had a 7-day history of vaginal bleeding. Ultrasonography and magnetic resonance imaging (MRI) showed an oval mass in the cervix, which was initially considered as neoplastic lesions and highly suspected to be cervical cancer, but cervical leiomyoma and other benign tumors cannot be completely excluded. Subsequently, cervical biopsy showed that the tumor was SEP, and then the patient underwent surgery. The postoperative pathological diagnosis was also SEP, which confirmed the radiologist’s misjudgment. In conclusion, SEP that occurs in the cervix is remarkably rare, and only nine cases have been reported in the cervix. No case reports to date have described in detail the imaging findings of cervical SEP. This study demonstrates the MRI imaging characteristics of a patient with SEP of the cervix and reviews the imaging findings of SEP reported in the previous literature, in order to provide more extensive insights for radiologists to consider the differential diagnosis of cervical lesions.

## Case Presentation

**Figure 1:**
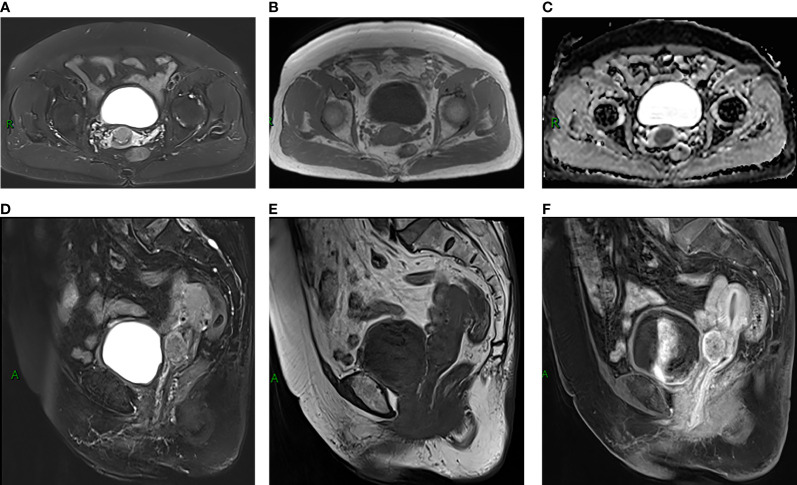
**(A, B, D, E)** MRI scans showed a well-defined oval mass located in cervix, which destroyed the stroma **(A)**. The mass showed slight hyperintensity on T2-weighted MR images **(A, D)** and isointensity on T1-weighted MR images **(B, E)**. **(C)** The ADC map showed low ADCs. **(F)** Sagittal contrast-enhanced MR image showed mild to moderate heterogeneous enhancement of the lesion, presenting rim-enhancement.

**Figure 2:**
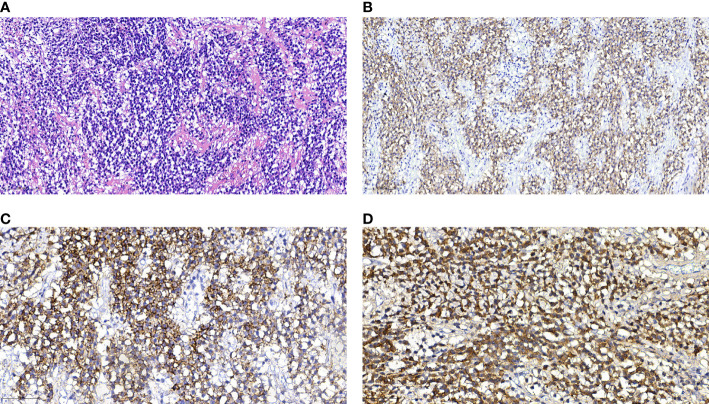
Histological and immunohistochemical features of SEP. **(A)** Hematoxylin-eosin staining shows that the monoclonal well-differentiated plasma cells are diffusely distributed and consistent in size. **(B–D)** Immunohistochemical staining presented CD38(+), CD138(+), Kappa (+). [Original magnifications: **(A, B)** 200×; **(C, D)** 400×].

A 77-year-old female patient was admitted to hospital because of a 7-day history of abnormal vaginal bleeding, with no abdominal pain or distention. Gynecological examination revealed cervical atrophy, and the normal shape of the cervix disappeared. There was no contact bleeding of the cervix and no tenderness of the posterior fornix. She had no other symptoms, and the results of the laboratory examinations were normal. Ultrasonography showed hypoechoic nodule in the cervix. The pelvic magnetic resonance imaging (MRI) scan found a well-defined oval mass of size 2.3×1.8×2.8 cm in the cervix, which destroyed the continuity of the cervical stroma **(****Figure 1A****)**. On MRI, the mass was homogeneous, slightly hyperintense on the T2-weighted images **(****Figures 1A, D****)**, and isointense on the T1-weighted images **(****Figures 1B, E****)**. Decreased apparent diffusion coefficient (ADC) value were detected in the cervical lesion **(****Figure 1C****)**. Post-contrast enhancement images showed mild to moderate heterogeneous enhancement and pronounced peripheral rim enhancement **(****Figure 1F****)**. According to the patient’s age, clinical manifestations and imaging features, the radiologist’s initial consideration was neoplastic lesions and the possibility of cervical cancer was high. The possibility of cervical leiomyoma cannot be fully ruled out, however. The patient accepted colposcopic examination and underwent cervical biopsy and endocervical curettage under colposcopic guidance. The biopsy specimens revealed SEP on microscopic examination. Additionally, bone scan revealed no abnormal skeletal uptake, and no abnormal plasma cells were found in the bone marrow, which excluded the diagnosis of multiple myeloma (MM). Following discussion, the clinicians decided that the best treatment strategy was surgery with postoperative radiotherapy. Then, the patient underwent a laparoscopic extensive hysterectomy, bilateral salpingo-oophorectomy, and pelvic lymph node dissection. The resected mass size was about 3.0×2.0 cm. The result of histopathological examination revealed a SEP of cervix. Immunohistochemical staining was positive with CD38, CD138, Kappa **(****Figure 2****)** and negative with Lambda. The Ki-67 proliferation index was low (5%). The patient received radiotherapy after surgery and remains well through follow-up.

## Discussion

SEP can occur in any part of the extramedullary sites but predominantly in the head and neck, particularly in the nasal cavity, sinuses, nasopharynx, and larynx (1). Other common sites are gastrointestinal tract, urogenital tract, skin, lung and breast (2). These areas are rich in lymphoid tissues and plasmacyte cells (3, 4). SP is more often found in male patients over 40 years of age (5). According to 9 previously reported cervical SEP cases (6, 7) and our case, the patients’ age ranges from 21 to 77 years old, and the mean age is 41 years old. The clinical manifestations are diverse, such as coital bleeding, vaginal discharge, vaginal bleeding, pelvic pain, etc. However, some patients are asymptomatic (6). Our patient presented with vaginal bleeding. Postmenopausal women with vaginal bleeding should first be excluded from common malignancies such as endometrial cancer and cervical cancer. In addition, other diseases such as endometrial polyps, submucous uterine fibroids and cervical polyps should also be given consideration, but SEP should also be considered, despite its rare occurrence. The computed tomography (CT) imaging finding of SEP is a low-attenuation mass with slight heterogeneity. On MRI, SEP usually manifests as a well-defined or ill-defined soft-tissue tumor with mass effect, showing iso/hypo-intense on the T1-weighted images and iso/hyper-intense on the T2-weighted images compared with muscles. The contrast-enhanced imaging is described as mild to marked heterogeneous enhancement on both CT and MRI (2, 8, 9). On diffusion weight imaging (DWI), SEP can show restricted diffusion, suggesting high cellularity in the lesion (10). Generally, the tumor rarely metastases to other sites but is locally invasive and can destroy, infiltrate or encroach adjacent structures (2, 8). Besides, necrosis may occur in larger tumors (2). By reviewing the literatures of SEP, we have not found the occurrence of hemorrhage, calcification, and fat (10). CT has greater advantages in evaluating bone destruction comparing to MRI (2).

In our case, the tumor showed slight hyperintensity on the T2-weighted images and isointensety on the T1-weighted images. The lesion was hyperintense on DWI and hypointense on ADC may indicate tumor cells are closely arranged. Following contrast enhancement, the tumor exhibited a mild to moderate inhomogeneous enhancement and the edge appeared more obviously, which may suggest that the blood supply was more abundant at the edge of the tumor. The tumor was oval with clear borders. It destroyed the cervical stroma, which indicated aggressive tumor behaviors. Some of the imaging manifestations of our case are similar to cervical leiomyoma and early cervical cancer. Uterine leiomyoma is benign tumor with well-defined borders and occurs primarily from the uterine body or less commonly from the cervix. The most specific manifestation of cervical leiomyoma is obviously low intensity on T2-weighted images (11). In this case, the signal intensity on T2-weighted images was evidently not low enough and the lesion was invasive, not consistent with the manifestations of typical leiomyoma. Cervical cancer often manifests as a round, lobulated or irregular soft tissue mass on MRI, showing iso**/**slight hypo-intensity on the T1-weighted images, medium hyper/hyper**-**intensity on the T2-weighted images, and obvious high signal on DWI with a corresponding low signal on ADC maps. The malignant progression of cervical cancer can spread to the vagina, parauterine tissues, adjacent organs, and distant metastasis. Of note, cervical cancer is a hypervascular tumor, in which the most significant imaging feature is early and obvious enhancement on the dynamic contrast enhanced MRI (DCE-MRI), but the late enhancement is lower than the normal cervical stroma (12).

SEP in the cervix needed to be distinguished from other lesions such as abscesses, lymphomas and rectal stromal tumors. Cervical abscess is not a common pelvic inflammatory disease (PID). The abscess cavity contains a fluid component and is surrounded by edema (13). The abscess typically presents as ring-like and/or septal enhancement (14). Lymphoma makes up 3.5% of all malignant tumors in women (15). Primary cervical lymphomas originate from the cervical stroma are characterized by rapid growth and rare necrosis (16, 17). Structure preservation and intact squamous epithelium are other features of them (16). The typical imaging appearance of cervical lymphoma has been described as a mass with homogeneous signal, unclear edge, and moderately homogeneous enhancement (16). Gastrointestinal stromal tumors (GISTs) are found in the gastrointestinal tract and need to be differentiated from cervical tumors when they occur in the rectum (18). The occurrence of rectal GISTs is more common in men aging from 50 to 60 years old (19). Rectal GISIs are characterized by an exophytic growth pattern, but anatomical continuity with the rectum can be observed on the imaging (19). Small GITIs (≤5cm) are usually round or oval, with homogeneously obvious and persistent enhancement, whereas large GITIs (>5cm) are lobulated with mild, inhomogeneous gradual enhancement, and cystic changes are often seen in the tumor (18). It has been reported that GISIs can appear calcification, mostly focal and patchy (19). However, definitive diagnosis requires histological examination, and imaging is of limited utility in differential diagnosis.

SEP is usually a localized low-grade malignant tumor, and the therapeutic purpose is mainly local control (20). Because SP is highly sensitive to radiation (1), most authors generally agree that radiotherapy as the standard treatment of SP and excellent local tumor control rates can be achieved. A radiotherapy dose of 40–50 Gy is recommended by most of the authors (the daily dose is 1.8-2 Gy), and the total treatment course is about 4 weeks (20). At present, the treatment of SP includes radiotherapy, surgery or a combination of both (4). The efficacy of chemotherapy in the treatment of SEP is controversial and further research is needed. Surgical resection may as an option for the treatment of SEP with clear surgical margins or those that occur in rare area with small size (20). However, some scholars consider that single surgery is not recommended even in the case of complete resection. The combination of surgery and radiotherapy can achieve better therapeutic effects and survival results (21). The lesion presented in this case is located in a rare part of the cervix, with a clear boundary and small size, so it is more appropriate to use surgery plus postoperative radiotherapy. While current clinical treatments for cervical cancer are mainly surgery, radiotherapy and chemotherapy. The major treatment strategy for early cervical cancer is surgery, and the treatment of middle and advanced cervical cancer is primarily radiotherapy supplemented with chemotherapy. However, patients with recurrent or metastatic cervix cancer have dismal prognosis. Therefore, early detection of cervix lesion and make a correct diagnosis would be extremely helpful to choose optimal therapy and improve the survival rate of patients.

The progression of SP includes the appearance of new plasmacytoma or the progression to MM. SBP is more likely to progress to MM than SEP (1). Risk factors for progression to MM include persistence of a serum monoclonal protein after treatment and abnormal serum SFLC ratio (< 0.26 or > 1.65). Furthermore, minimal bone marrow plasmacytosis is a contributing factor to decreased progression-free survival. Furthermore, some authors identified age (≤60 years), tumor size (<4 cm) and location as prognostic factors of SP (21). Generally speaking, the current research and trials on SP are very limited, let alone SP in uncommon sites, therefore it is necessary to observe the recurrence or metastasis of patients through follow-up.

In conclusion, we present an extremely rare case of SEP in the cervix and its characterization on MRI. SEP should be considered in the differential diagnosis of cervical lesions without a history of multiple myeloma.

## Data Availability Statement

The raw data supporting the conclusions of this article will be made available by the authors, without undue reservation.

## Ethics Statement

Written informed consent was obtained from the patient for the publication of this case report.

## Author Contributions

JW and LJ: manuscript writing. TL: pathological review. XM, XC, and HL: manuscript revision. SL: conception and critical review. All authors contributed to the article and approved the submitted version.

## Conflict of Interest

The authors declare that the research was conducted in the absence of any commercial or financial relationships that could be construed as a potential conflict of interest.
